# Influence of Sunlight Incidence and Fruit Chemical Features on Oviposition Site Selection in Mango by *Anastrepha obliqua*: Implications for Management

**DOI:** 10.3390/insects13020141

**Published:** 2022-01-28

**Authors:** Larissa Guillén, Juan L. Monribot-Villanueva, José A. Guerrero-Analco, Rafael Ortega, Alma Altúzar-Molina, Victoria Mena, Eliel Ruiz-May, Martín Aluja

**Affiliations:** 1 Instituto de Ecología, A.C. (INECOL)—Clúster Científico y Tecnológico BioMimic^®^, Red de Manejo Biorracional de Plagas y Vectores, Carretera Antigua a Coatepec 351, El Haya, Xalapa 91073, Veracruz, Mexico; rafael.ortega@inecol.mx (R.O.); alma.altuzar@inecol.mx (A.A.-M.); martin.aluja@inecol.mx (M.A.); 2 Instituto de Ecología, A.C. (INECOL)—Clúster Científico y Tecnológico BioMimic^®^, Red de Estudios Moleculares Avanzados, Carretera Antigua a Coatepec 351, El Haya, Xalapa 91073, Veracruz, Mexico; eliel.ruiz@inecol.mx; 3 Instituto Tecnológico de Úrsulo Galván, Carretera Cardel—Chachalacas Km 4.5, Úrsulo Galván 91667, Veracruz, Mexico; agami_virgo2@hotmail.com

**Keywords:** Tephritidae, *Mangifera indica*, sunlight-effect/oviposition decisions, untargeted plant metabolomics, mangiferin, abscisic acid

## Abstract

**Simple Summary:**

The mango fruit fly, *Anastrepha obliqua,* is one of the most important pests attacking mangos in Mexico and Central and South America. With the aim of identifying key factors that could help to better control/manage this pest, we determined the preferred sites in the fruit where *A. obliqua* females lay their eggs, registering if these sites were in the upper, middle, or lower sections of the fruit, if they were exposed to sunlight incidence, and if they had special concentrations of some nutritional and chemical compounds. Females mainly oviposited in shaded, upper fruit sections where the pulp was richer in carbohydrates than non-oviposited sections and, where mangiferin, a polyphenol known for its antioxidant properties, was in higher concentrations. The absence of abscisic acid (ABA) and dihydrophaseic acid glucoside, a by-product of ABA catabolism in non-oviposited sections, suggests that this chemical compound could play a role in fruit acceptance behaviors by female flies. We conclude that *A. obliqua* females prefer ovipositing in fruit sections, where fruit environmental and chemical conditions are optimal for egg and larval development and propose a management scheme directly based on this information.

**Abstract:**

With the aim of identifying key factors that determine oviposition decisions by *Anastrepha obliqua* for management purposes, we conducted a behavioral study under natural/semi-natural field conditions to identify where exactly in the fruit (upper, middle, or lower sections) females preferred to lay eggs in a highly susceptible mango cultivar (“Criollo”), and whether sunlight incidence and fruit chemical compounds influenced oviposition site selection by this pestiferous fly. Females oviposited in shaded, upper fruit sections where pulp had higher total carbohydrate concentrations but similar total protein, lipid, and polyphenol concentrations than non-oviposited sections. Peel had higher overall nutrient and mangiferin/quercetin-3-D-galactoside (polyphenols) concentrations. An untargeted metabolomic analysis of oviposited and non-oviposited fruit sections identified abscisic acid (ABA) and dihydrophaseic acid glucoside, a by-product of ABA catabolism, as potential chemical markers that could play a role in fruit acceptance behaviors by female flies. We conclude that females preferentially oviposit in fruit sections with optimal chemical and environmental conditions for larval development: more carbohydrates and antioxidants such as mangiferin and ferulic acid and lesser sunlight exposure to avoid lethal egg/larval desiccation/overheating. We make specific recommendations for *A*. *obliqua* management based on female host selection behavior, a tree pruning scheme exposing fruit to direct sunlight, application of a host marking pheromone, and the use of egg sinks in the orchard.

## 1. Introduction

To successfully manage a pest taking advantage of vulnerable aspects/stages in its biological cycle, behavior, or population dynamics, field studies are critical [1,2,3,4]. For example, mating disruption [5,6], attract and kill [7], push-pull [8,9], trap cropping [10,11,12], and other environmental manipulation strategies for pest control [13,14], all hinge on a deep understanding of the behavior of the pest [1,15]. In the case of fruit flies (Diptera: Tephritidae), the behavior of females is particularly relevant as they are the ones that lay eggs inside fruit, causing severe economic damage when larvae start to feed on the valuable pulp [16]. Therefore, any insight gained on the factors that regulate/drive female oviposition decisions and what external abiotic (e.g., ambient temperature and humidity, incidence of sunlight/light intensity) or biotic factors (e.g., fruit chemistry) impinge the most on exactly where eggs are laid, will significantly improve our chances to efficiently control/manage the pest.

To understand the factors that guide fruit fly (Diptera: Tephritidae) females during the catenary process of oviposition site selection once on a tree/fruit sensu [17,18], aspects such as fly age and egg load, aculeus wear, presence of conspecifics or predators, and host-quality, among others, have been studied [16,19,20,21]. Females can use fruit physical and chemical features from the host, such as sugar content, presence of toxic allelochemicals, and physical barriers (e.g., surface waxes, the width of the cuticle) to evaluate host quality [20,22,23,24,25]. Since progeny survival is closely correlated with fruit quality, the selection of suitable hosts for larval development by females is critical for the reproductive success of these flies [26]. The process of optimal host selection is further complicated because physical and chemical properties do not only vary among fruits in a tree, but homogeneity can also be measured in different parts of a single fruit [27,28,29].

The West-Indian or mango fruit fly, *Anastrepha obliqua* (Macquart), is one of the most important mango (*Mangifera indica* L.) pests in Mexico, Central and South America [30,31,32]. Females exhibit short life spans (ca. 45 days) and lay a single egg per oviposition bout in host fruit, usually distributed discretely in time and space [32]. Its native hosts, the tropical plum, *Spondias purpurea* L. and *S. mombin* L. (both, Sapindales: Anacardiaceae), can simultaneously bear between 2000 and 20,000 fruits in short periods of three to five weeks [33]. This abundance of oviposition resources can result in periods of egg-limitation in *A. obliqua* females [34]. Since in egg-limited species, females run out of eggs before oviposition opportunities decline or disappear [35], females can be more selective in their oviposition decisions than time-limited species [34,36]. Under laboratory conditions, *A. obliqua* females choose to oviposit into more nutritious substrates when given a choice (i.e., agar spheres with added protein or without protein) [37] and avoid ovipositing into toxic substrates (agar spheres with citrus flavonoids), which affect the fitness of larvae and pupae [38].

*Anastrepha obliqua* females have a short aculeus (1.4–1.7 mm long), and when they oviposit into mangos (a much larger fruit than the ancestral hosts *Spondias* spp.), eggs are often laid in the peel (where physical and chemical barriers are abundant [25]), exposing the respiratory horn, a structure critical for gaseous exchange, to desiccation if eggs are laid in fruit exposed to direct sunlight [39]. In addition, previous studies have clearly documented different degrees of mango susceptibility to the attack of *A. obliqua* related to cultivar type [40]. According to Guillén et al. [25], this susceptibility can be associated with the amount of resin ducts and sap content which can influence infestation levels and affect the development of immature stages of *A. obliqua* and *Anastrepha ludens* Loew (Diptera: Tephritidae). However, the high variability of fruit cuticle morphology among different mango cultivars [41], and mango-fruit chemical properties, could also explain why some cultivars such as “Criollo” are more susceptible to fruit fly attack than others such as “Tommy Atkins”, “Kent”, and “Ataulfo” [40].

Mango peel (cuticle) and pulp contain pectin, phenolics, tannins, carotenoids, proteins, and minerals [42,43], which may have favorable or unfavorable effects on insects. In the case of phenolics, they can have negative, neutral, or positive effects [44]. Phenolic compound concentrations can vary in a plant as a defense response to the attack of microorganisms, insects, or exposure to environmental factors such as light intensity [45,46]. In climacteric fruit such as mangos, where the plant hormone ethylene stimulates the ripening process of fruit, phenolic compounds such as flavonoids (influencing color, aroma, astringency, and antioxidant properties), carotenoids and soluble solids such as sugars, among other chemicals, are affected by sunlight exposure [47,48,49] and by natural physiological processes during development [50].

Some phenolic compounds reported in mangos are also present in apples where they inhibit feeding and interfere with the process of metamorphosis of *A. ludens*, a close relative of *A. obliqua*, to the extent that apple cultivars with high levels of phenolic compounds were shown to be totally resistant to fruit fly attack [40]. A situation where mango cultivars with high phenolic compound concentrations were less infested than fruit with low concentrations [51] was observed. This was also seen in the oriental fruit fly, *Bactrocera dorsalis* (Hendel) (Diptera: Tephritidae).

In addition to fruit features, microclimatic factors such as temperature, relative humidity, and light intensity can also influence host quality perception by female flies [52] or behavior [53]. Aluja and Birke [54] reported that most of the oviposition events of *A. obliqua* in the field occurred when sunlight intensity and temperature were lowest (four footcandles and 27 °C, respectively), and that oviposition activity dropped when the sunlight intensity and temperature was between 14–32 footcandles and 34–40 °C, respectively. In *Bactrocera oleae* (Rossi), the olive fly, Kokkari et al. [55] found that females produce a similar number of eggs at low (20 Lux) or high (1600 Lux) light intensity in the presence of fruit. In other insects, it has been shown that solar ultraviolet-B radiation can affect oviposition behavior [56].

There are few studies on the interaction of sunlight incidence on fruit, the fruit’s chemical properties, and the effect of both on a phytophagous insect’s behavior. There are also few studies concerning: (1) oviposition decisions by females in insects with larvae that cannot move to another plant or fruit, which determines if progeny will survive and greatly influences various fitness parameters [26,57], (2) fruit features that are heterogeneous in their spatial distribution within a fruit [20,28,29], and (3) plants that can modify defense chemicals to protect themselves from environmental factors such as sunlight. Using *A. obliqua* as a model system, we explored the combined effect of sunlight and chemicals in “Criollo” mangos on the specific site in fruit where *A. obliqua* females lay their eggs. Studies on host selection by fruit fly females have focused on comparing broad/general chemical and physical fruit features but have not considered the influence of variable fruit chemistry in combination with microclimatic factors such as light incidence on the fruit on oviposition decisions by females. The only exception is Rattanapun et al. [20], who observed the oviposition preferences of *B. dorsalis* in different parts of mangos with different degrees of ripening and associated these preferences with larval development time, pupal weight, adult emergence, and body size. Expanding on the “preference-performance” hypothesis [57], which predicts that adult females select hosts with optimal features to lay their eggs to maximize offspring fitness, and the related “mother-knows-best” hypotheses, which predicts that mothers modulate oviposition decisions so as to optimize offspring survival [26,58,59], we concentrated on oviposition decisions by females per se, not their outcome (i.e., larval survival, concomitant adult fitness), and measured fruit quality parameters likely influencing these decisions. Our specific aims were to determine: (1) whether concentrations of phenolic compounds in a mango fruit are the same in areas exposed to sunlight or in shaded areas, (2) whether the sites selected by *A. obliqua* females to oviposit into a “Criollo” mango are chemically different when compared to fruit parts left untouched, and (3) whether both chemical fruit features and sunlight condition can explain *A. obliqua* female oviposition site selection in a highly susceptible mango cultivar such as “Criollo”. We predicted that females would reject most sections of the fruit to lay their eggs, and that the chosen sites to oviposit would exhibit optimal conditions for egg and larval survival/development (e.g., ideal sunlight, lower polyphenol and higher nutrient concentrations). We also predicted that females would clump eggs by repeatedly inserting the aculeus/eggs in the neighborhood of previously used oviposition sites (*A. obliqua* females only lay one egg per oviposition bout).

## 2. Materials and Methods

### 2.1. Study Insects

*Anastrepha obliqua* flies stemmed from naturally infested “Criollo” mangos collected in Tolome, Veracruz, Mexico (19°16′26.8″ N, 96°23′24.34″ W). The infested fruit were transported to the laboratories of the Red de Manejo Biorracional de Plagas y Vectores (RMBPV) laboratory at the Clúster Científico y Tecnológico BioMimic^®^ (Mexico City, Mexico) of the Instituto de Ecología, A.C. and processed and placed in perforated plastic trays on top of plastic washbowls containing vermiculite as a pupation medium to obtain adult flies. Every third day, vermiculite was inspected, and recovered pupae placed in a 250 mL plastic container with moist vermiculite, covered with a muslin lid, and kept at 27 ± 1 °C, 75 ± 5% r.h. until fly emergence. Groups of 30 females and 15 males of recently emerged *A. obliqua* flies were placed in 30 × 30 × 30 cm Plexiglas cages and fed *ad libitum* with a 3:1 mixture of sugar and hydrolysed yeast and a piece of water-soaked cotton until adults reached sexual maturity. Cages with flies were kept at 27 ± 1 °C, 75 ± 5% r.h., and a photoperiod of L12:12D until their transport to the field.

### 2.2. Study Sites

The oviposition studies were performed in two “Criollo” cultivar mango orchards located in Cardel (19°22′ N, 96°22′ W; altitude 20 masl) and Actopan (19°41′ N, 96°52′ W; altitude 100 masl), Veracruz State, Mexico. The mean annual temperature in this region is 24.8 °C with a mean rainfall of 1178.5 mm in Cardel and 860 mm in Actopan (SMN, 2020). In both sites, the rainy season lasts 4.6 months (July to October) and the dry season lasts 7.4 months (October to May).

### 2.3. Oviposition under Semi-Natural Field Conditions and Fruit Chemical Analyses

#### 2.3.1. Study 1: Oviposition Observations and Forced Fruit Infestation

**Figure 1:**
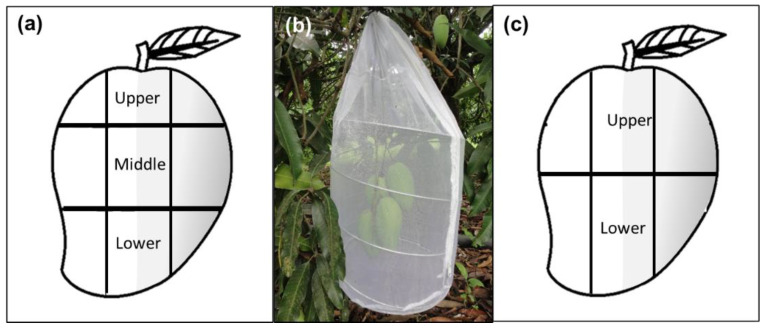
Sampling approaches and branch-bagging scheme followed depicting sections (upper, middle and/or lower) and shaded (gray color) or sunny areas used in the experiments under semi-natural field conditions: (**a**) Sampling design used in the studies of oviposition behavior, infestation, determination of total phenolic compounds, proteins, carbohydrates, and lipids in Studies 1 and 2; (**b**) Branches with marked mango fruit bagged with an organza cage for observations on female’s oviposition activity; (**c**) Sampling design used for the targeted and untargeted metabolomic analysis of fruit phenolic compounds in Studies 3 and 4.

Approximately one month prior to field observations (i.e., May), three branches with at least five unripe fruits from five different “Criollo” mango trees were bagged with white chiffon bags (1.26 × 0.61 m) to prevent natural fruit fly infestation and damage by other insects or pathogens. When fruit reached the green-ripe stage (a ripening stage ideal for female oviposition activity) one day before observations, some leaves close to the fruit were removed to improve visibility, and to mark mango sections. Each fruit was divided into three sections (upper, middle, and lower) with a green water-based marker (Figure 1a). Each section was then equivalently divided into five squares, yielding a total of 15 squares per fruit. Squares were numbered from 1 to 15 (1–5 upper section, 6–10 middle section, 11–15 lower section) and marked to further control which “square numbers” were sun-exposed or not. Then, each fruit was additionally labeled with a numbered ribbon attached to the peduncle. Branches with marked fruit were covered with a white organza cylindrical cage (0.33 m diameter × 0.75 m long) (Figure 1b). The next day, 30 min before observations started, fifteen 17–19-day old, mated *A. obliqua* females and five males (included in case a female had not mated in the laboratory) were released into the cage (ratio of three females and one male per fruit). Observations involving one branch/cage per day were carried out continuously during eight hours from 8:00 to 16:00 h. The next day, a different branch/cage was selected for observations. For each numbered square in every fruit, the number of ovipositions were registered. Flies were left inside the cylindrical cages with fruit for two more days for additional ovipositions. The fruit were then harvested, labeled individually, placed in 1 L plastic containers with a thin layer of vermiculite, and kept under laboratory-controlled conditions at 26 ± 1 °C and 75 ± 5% r.h. After ten days, the fruit were carefully dissected, and the number of larvae per square and section was recorded.

#### 2.3.2. Study 2: Oviposition Observations and Fruit Chemical Analysis

For oviposition observations under field conditions, the same methodology described in the previous section was used. The number of effective ovipositions (i.e., followed by aculeus dragging and deployment of the host marking pheromone [HMP]) per fruit square and section was recorded, registering if light impinged on the fruit or not (i.e., shaded and sun-exposed fruit sides). At the end of the observation period, marked fruit were removed, placed inside coolers, and transported to the laboratory to process and quantify the total phenolic compounds and protein/lipid/carbohydrate content. Temperature and relative humidity were registered each hour.

##### Total Nutrient and Phenolic Content Analyses

For the analyses of nutrients (proteins, lipids, and carbohydrates), and total phenolic compounds, 30 fruit were immediately processed after arrival from the field. Based on behavioral observations, two oviposited (one for sunny and one for shaded areas) and two non-oviposited (one for sunny and one for shaded areas) mango squares were cut out from each replicate fruit (N = 30) with a dissection knife. Each square was separated by peel and pulp (mesocarp), then chopped, placed on a piece of aluminum foil, weighed using an electronic balance (Ohaus^®^, Mexico City, Mexico), labeled, immersed in liquid nitrogen, and stored at −80 °C until chemical analyses.

- Total phenolic compounds. The extraction of total phenolic compounds was performed following the procedures described by Singleton et al. [60] and adapted by Konelab ARENA 20XT (Thermo Fisher Scientific OY, Vantaa, Finland). Aliquots of 100 mg of sample were placed in 1.2 mL Qiagen microtubes containing one ml of methanol (J.T. Baker, PN: 9093-03, both from, New York, NY, USA) with a 0.3 mm diameter stainless steel pearl and homogenized in a Tissue Lyser II (Qiagen^®^, Hilden, Germany) for 10 min at 30 Hz frequency. Macerated samples were allowed to stand for one h at room temperature. Then, 800 µL aliquots were placed in new 1.5 mL Eppendorf tubes and centrifuged at 3000 rpm for one min in a centrifuge HETTICH D-78532 (Tuttlingen, Germany). Once centrifuged, 600 µL of supernatant was placed in a new 1.5 mL Eppendorf tube. Duplicate aliquots of 10 µL of the supernatant of each sample or standard (different concentrations of gallic acid [Sigma-Aldrich, PN: G7384, St. Louis, MO, USA] in methanol [J. T. Baker, PN: 9093-03, both from, NJ, USA]) were placed in the wells of a 96-well microplate with 10 µL of Folin–Ciocalteu (Sigma-Aldrich, PN: F9252, St. Louis, MO, USA) and 100 µL of distilled water added to each well and then allowed to stand for one min at room temperature. Then, 40 µL of 7% sodium carbonate (Sigma-Aldrich, PN: S7795, St. Louis, MO, USA) aqueous solution and 40 µL of distilled water were added to fill each well to 200 µL. Samples and standards were heated at 37 °C for five min and then read in a Microplate Spectrophotometer (EPOCH 2TC, BioTek VT, USA) fitted with a 700 nm filter. For quantification, the calibration curve with different standard (gallic acid) concentrations 0, 0.025, 0.05, 0.1, 0.2, 0.4, 0.6, and 0.8 mg/mL) was used as a reference.
- Total protein concentration. For total protein extraction, aliquots of 100 mg of frozen samples (pulp or peel) with one ml of phosphate-buffered saline (PBS; pH 7.0; Sigma-Aldrich, PN: P3813, St. Louis, MO, USA) and a 0.3 mm diameter stainless steel pearl were placed into a 1.2 mL Qiagen microtube and homogenized in a Tissue Lyser II (Qiagen^®^, Hilden, Germany) for 10 min at 30 Hz. Thereafter, one ml of PBS buffer was added, and samples were shaken for two minutes. All samples were transferred to new 1.5 mL tubes and centrifuged at 3000 rpm for five min. Once centrifuged, the supernatant of each sample was placed in a new 1.5 mL microtube and centrifuged at 13,000 rpm for four min. For protein quantification, we used the Pierce BCA Protein Assay kit (Sigma-Aldrich, PN: QPBCA, St. Louis, MO, USA) and the bovine serum albumin (BSA; Sigma-Aldrich, PN: A9418, St. Louis, MO, USA) as standard. A 540 nm filter was used to read samples and standards absorbances in the Microplate Spectrophotometer (EPOCH2TC, Biotek, VT, USA). Protein quantification was calculated by comparing sample values with the BSA standard curve (0, 1, 2, 3, 4, 5, 6 and 7 mg/mL).
- Total lipid and carbohydrate determinations. For total lipid and carbohydrate extraction, we used adaptations of methodologies reported by Warburg and Yuval [61], Yuval et al. [62], and Nestel et al. [63]. Ten mg samples of frozen pulp or peel were placed in a porcelain dish with 200 µL of 2% sodium sulfate (Sigma-Aldrich, PN: 239313, St. Louis, MO, USA) solution and then homogenized using a small pestle. Each sample was then placed in a 1.5 mL tube. Residues from the dish were recovered by rinsing tubes twice with 500 µL of chloroform methanol (1:2) (chloroform, PN: 15,598,554 and methanol GC, PN: 9093-03, both from J. T. Baker, New York, NY, USA) and added to the sample in the tube. Samples were shaken and centrifuged at 10,000 rpm for 10 min in a centrifuge (HETTICH D-78532, Tuttlingen, Germany). A 300 µL volume of the supernatant was then placed in a new 1.5 mL tube for lipid analysis, and the remainder of the sample was kept in a refrigerator at 4 °C for carbohydrate analysis. Lipid samples and a set of different triolein (Sigma-Aldrich, PN: 44895-U, St. Louis, MO, USA) concentrations as quantification standards (0, 50, 100, 150, 200, 250, 300 µg/mL) were incubated at 75 °C for 30 min, then at 95 °C until total liquid evaporation (ca. 20 min). A 300 μL volume of sulfuric acid (Sigma-Aldrich, PN: 357413, St. Louis, MO, USA) was added to each sample and standard, vortexed and incubated at 100 °C for 10 min. Samples and standards were then allowed to cool. Then, 30 µL of samples were placed in a 96 well microplate, 270 µL of vanillin (Sigma-Aldrich, PN: V1104, St. Louis, MO, USA) reagent (600 mg of vanillin dissolved in 100 mL of distilled water and 400 mL of 85% of H_3_PO_4_ [Sigma-Aldrich, PN: P5811, St. Louis, MO, USA]) were added, and incubated for 25 min at room temperature. After this, samples and standards were read in the Microplate Spectrophotometer (EPOCH2TC, Biotek, VT, USA) fitted with a 492 nm filter. Lipid quantification was estimated by comparing sample values with the standard curve. For analysis of carbohydrates, a 300 µL volume of the supernatant used for lipid analysis was incubated at 75 °C until all liquid had evaporated. Then, 400 µL distilled water was added, vortexed, and 50 µL of this mixture plus 150 µL distilled water was placed in a new 1.5 mL tube. Simultaneously, a range of different concentrations of glucose (0, 5, 10, 15, 20, 25, 30 µg/µL; Sigma-Aldrich, PN: G8270, St. Louis, MO, USA) with distilled water was prepared to generate a calibration curve. Then, one mL of anthrone (Sigma-Aldrich, PN: 319899, St. Louis, MO, USA) reagent (500 mg of anthrone dissolved in 500 mL of sulfuric acid [Sigma-Aldrich, PN: 357413, St. Louis, MO, USA]) was added to samples and standards, vortexed, and heated at 90 °C for 10 min. Aliquots of 300 µL of samples and standards were then placed in a 96 microplate and read in the Microplate Spectrophotometer (EPOCH2TC, Biotek, VT, USA) at 630 nm. Carbohydrate quantification was performed by comparing sample values with the standard curve.

### 2.4. Study 3: Untargeted Metabolomic Analysis of Mangos

Forced oviposition bioassays were ran in the locality of Naranjos, Puente Nacional, Veracruz, Mexico (19°21′18″ N, 96°31′49″ W; altitude 100 masl) in 40 “Criollo” mangos from four trees (10 from each one). Forty control fruit (not exposed to the oviposition activity of *A*. *obliqua* females) were also collected in neighboring branches of the same trees. We allowed 15–20-day-old mated *A. obliqua* females to lay eggs into the preferred area between the upper and upper/middle parts of mangos (Figure 1c). Then, we collected 200 mg of skin and pulp in the exact location where the single egg had been oviposited with a 0.5 cm diameter stainless steel punch tool inserted at 0.5 cm depth. We proceeded identically in the case of control fruit devoid of eggs, considering the same fruit sections as oviposited samples. The samples (pulp and skin) were immediately transferred to a labeled 1.5 mL vial and frozen with liquid nitrogen. Once back in the laboratory, the samples were transferred to a −80 °C ultra-freezer until processing. The first metabolomic analyses we ran considered 15 individual mango samples. Based on the results obtained, we ran a complimentary analysis, using pools of six mangos per sample for each condition (i.e., fruit with eggs or devoid of them). Each sample was injected in a Waters Class I UPLC coupled to a high-resolution mass spectrometer (Synapt G2 Si, Waters, Milford, MA, USA). Prior to injections, samples were dried in a Labconco freeze-dryer, and methanolic (MS grade, Honeywell PN: 34966-4L, Seelze, Germany) extracts were obtained using an accelerated solvent extraction system (ASE 350, Thermo Scientific, Waltham, MA, USA) as described previously [64]. The extracts were filtered in 0.2 µm PTFE membranes (Agilent PN: 5191–5912, Santa Clara, CA, USA), and placed in 1.5 mL vials, and then injected in the UPCL-MS. Chromatography was carried out using an Acquity BEH column (1.7 µm, 2.1 × 50 mm; Waters PN: 186002350, Milford, MA, USA) with a column and sample temperatures of 40 and 15 °C, respectively. The mobile phase consisted of (A) water (MS grade, Honeywell PN: 14281-2L, Seelze, Germany) and (B) acetonitrile (MS grade, Fisher Chemical PN: LS120-4, Geel, Belgium), both with 0.1% formic acid (*v*/*v*). The gradient conditions of the mobile phases were 0–13 min linear gradient 1–80% B, 13–14 min 80% B isocratic, 14–15 min linear gradient 80–1% B (total run time 20 min). The flow rate was 0.3 mL/min and one µL of each extract was injected. The mass spectrometric analysis was performed with an electrospray ionization source in positive mode with a capillary, sampling cone, and source offset voltages of 3000, 40, and 80 V, respectively. The source temperature was 100 °C and the desolvation temperature was 20 °C. The desolvation gas flow was 600 L/h, and the nebulizer pressure was 6.5 Bar. Leucine-enkephalin was used as the lock mass (556.2771, [M+H]^+^). The conditions used for MS^E^ analysis (acquisition method of Waters™ company) were mass range 50–1200 Da, Function 1 CE, 6 V, function 2 CER 10–30 V, and scan time 0.5 sec. The data were acquired and processed with MassLynx (version 4.1, Waters, Milford, MA, USA). The tentative identifications were performed through comparison of the mass spectra (*m*/*z* values of molecular ions, adducts, and fragments) with those reported in public databases such as Metlin (https://metlin.scripps.edu/landing_page.php?pgcontent=mainPage, accessed on 29 October 2021) and Massbank (https://massbank.eu/MassBank/, accessed on 29 October 2021). The maximum mass error allowed was 5 ppm.

### 2.5. Study 4: Targeted Metabolomic Analysis of Plant Phenolic Compounds

Fruit material extraction. “Criollo” mangos not exposed to oviposition activity by *A. obliqua* females were divided into upper and lower sections, considering the sunny and shaded sides (Figure 1c). Three peel and pulp samples for each section were collected, frozen, and lyophilized (Freezone, Labconco, Kansas, MO, USA). Crude extracts of “Criollo” mango peel and pulp were prepared separately using methanol (MS grade, Honeywell PN: 34966-4L, Seelze, Germany) and an accelerated solvent extraction system (ASE 350, Thermo Scientific, Waltham, MA, USA) as described previously [64]. For peel, one ml aliquots of each sample were placed in 1.5 mL centrifuged tubes, and formic acid (MS grade, Sigma-Aldrich PN: 00940, St. Louis, MO, USA) was added to a final concentration of 0.1% (*v*/*v*). For pulp, 10 mL volumes were concentrated to dryness using a rotary evaporator (Büchi RII, Büchi, Switzerland) under reduced pressure at 40 °C. A 50 mg sample of the dry extract was re-dissolved in one ml methanol with 0.1% formic acid (*v*/*v*) (both MS grade, Sigma-Aldrich, St. Louis, MO, USA). Finally, the peel and pulp were filtered in 0.2 µm PTFE membranes (Agilent PN: 5191–5912, Santa Clara, CA, USA) and placed in 1.5 mL UPLC vials for LC-MS analysis in a 1290 UPLC coupled to a 6460 triple quadrupole mass spectrometer (Agilent, Santa Clara, CA, USA). The identification and quantitation of individual phenolic compounds were performed as described by Juárez-Trujillo et al. [64] and expressed in µg/g of dry weight. Chromatography was carried out using a Zorvax SB-C18 column (1.8 µm, 2.1 × 50 mm; Agilent PN: 827700-902, Santa Clara, CA, USA) with a column and sample temperatures of 40 and 15 °C, respectively. The mobile phase consisted of (A) water (MS grade, Honeywell PN: 14281-2L, Seelze, Germany) and (B) acetonitrile (MS grade, Fisher Chemical PN: LS120-4, Geel, Belgium), both with 0.1% formic acid (*v*/*v*) (Sigma-Aldrich). The gradient conditions of the mobile phases were 0–40 min linear gradient 1–40% B, 40.1–42 min linear gradient 40–90% B, 42.1–44 min 90% B isocratic, and 44.1–46 min linear gradient 90–1% B (total run time 46 min). The flow rate was 0.1 mL/min and one µL of each extract was injected. The mass spectrometric analysis was performed with an electrospray ionization source in positive and negative modes. The desolvation and sheath gas temperatures were 300 and 250 °C, respectively. The nebulizer pressure was 45 psi. The cone gas (N_2_) and sheath gas flows were 5 and 11 L/min, respectively. The capillary and nozzle voltages were 3500 and 500 V, respectively. Authentic commercial standards were purchased for identification. (+)-Catechin (PN: C1251), mangiferin (PN: 06279), quercetin (PN: Q4951), quercetin-3-D-galactoside (PN: 83388) and quercetin-3-glucoside (PN: 17793) were purchased from Sigma-Aldrich (St. Louis, MO, USA). Caffeic acid (PN: 6018), ferulic acid (PN: 6077), protocatechuic acid (PN: 6050) and vanillin (PN: 6110S) were purchased from Extrasynthese (Lyon, France). A dynamic multiple reaction monitoring method was developed using the next transitions/retention times: protocatechuic acid 153 > 109.1 Da/6.4 min, (+)-catechin 169 > 93.03 Da/11.2 min, caffeic acid 181.04 > 163.03 Da/12.24 min, vanillin 153 > 124.9 Da/14.99 min, mangiferin 423 > 302.8 Da/15.18 min, ferulic acid 195.1 > 145.02 Da/19.18 min, quercetin-3-D-galactoside 465 > 303 Da/20.6 min quercetin-3-glucoside 465 > 303 Da/21.19 min and quercetin 302.9 > 153.1 Da/29.7 min. Calibration curves were constructed from 1 to 17 µM and quadratic type regressions were applied (r^2^ = 0.99). The data were acquired and processed with the MassHunter Workstation version B.06.00 (Agilent, Santa Clara, CA, USA).

### 2.6. Statistical Analyses

The number of ovipositions recorded in Study 1 was analyzed by fitting a Generalized Linear Model (GLM) with a Poisson error distribution, considering as predictor variable fruit section (upper, middle and lower) and as response variable the number of ovipositions per square. A Mann–Whitney U test was performed to compare the number of ovipositions per square according to sunlight incidence (shaded and sunny) on the fruit. The number of larvae of Study 1 was compared with a GLM model using a nested ANOVA, where the predictor variable, fruit section was nested in fruit and the response variable was the number of larvae per square. Total phenolic compounds and protein concentrations of Study 2 were analyzed in two ways: (1) with a factorial analysis of variance (ANOVA) considering as predictor variable fruit section (upper, middle and lower), light incidence (shaded and sunny) and fruit tissue (peel and pulp); (2) using t-tests to compare concentrations of total phenolic compounds or proteins in peel and pulp using as predictor variable the oviposited condition (oviposited or not-oviposited squares). The total lipid and carbohydrate concentrations of Study 2 were analyzed using a factorial analysis of variance (ANOVA) considering as predictor variables fruit tissue (pulp and peel) and oviposited condition (oviposited and non-oviposited squares). The statistical analyses related to the untargeted metabolomics data of Study 3 were performed with the MarkerLynx software (version 4.1, Waters, Milford, MA, USA) to identify discriminant chemical markers. We performed a principal component analysis (PCA) to distinguish between oviposited and non-oviposited samples. First, considering individual samples (*n* = 15 per treatment) and then pooled samples (*n* = 4 per treatment). Grouping in PCA figures is presented to show the location of samples per treatment. After that, we performed an orthogonal partial least-square discriminant analysis (OPLS-DA). The retention times and the protonated masses were generated at a noise threshold of 10,000 counts, and smoothing was applied. Pareto scaling was applied to generate the score plots. The variables that contributed to discrimination between the two groups were considered potential biomarkers in the S-plots. The specific phenolic compound concentrations of Study 4 (targeted metabolomic study) were analyzed in two ways: (1) using a nested MANOVA considering all the independent variables (fruit tissue, light incidence, and section (upper and lower), nesting, fruit section in light incidence) and considering as dependent variables the concentrations of the nine phenolic compounds measured; (2) via a nested ANOVA of the effect of section (nested according to light incidence) and light incidence on each concentration of phenolic compounds in the pulp or peel separately. Analyses were performed using the Statistica 7.0 program (Statsoft Inc., Tulsa, OK, USA).

## 3. Results

### 3.1. Female Oviposition Activity in “Criollo” Mango under Semi-Natural Field Conditions

**Figure 2:**
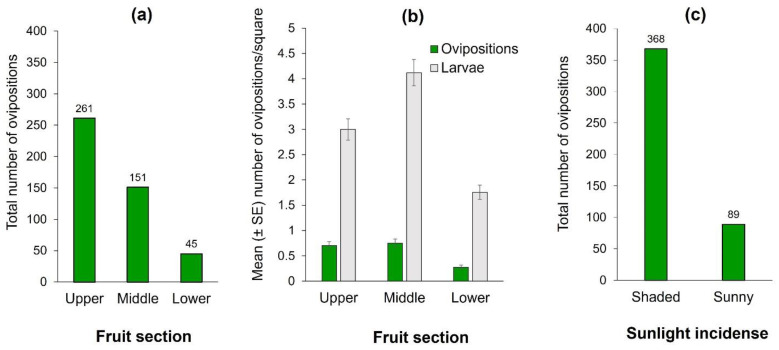
Ovipositions and infestation of *Anastrepha obliqua* on 75 “Criollo” mango fruit of Study 1. (**a**) Total number of *A. obliqua* ovipositions by fruit section; (**b**) Number of *A. obliqua* ovipositions and larvae per square according to fruit section (upper, middle, or bottom sections) in ‘Criollo’ mangos (mean ± SE) independent of sunlight incidence into fruit (i.e., shaded or sunny parts). Oviposition records correspond to the mean of observations conducted over an eight hour/day period. Infestations correspond to the mean of two days of natural infestation (i.e., mangos exposed to female oviposition activity); (**c**) Ovipositions according to sunlight incidence on fruit.

The number of ovipositions differed significantly among fruit sections (Wald X^2^ = 14.09, df = 2, *p* < 0.0001). The upper section received the highest cumulative number of observed ovipositions (Figure 2a). This result was coincident with the number of larvae, since the upper and middle sections had significantly more larvae than the lower mango section (Wald X^2^ = 167.48, df = 2, *p* < 0.0001) (Figure 2b). Clearly, flies clumped ovipositions in some squares and avoided laying eggs in others. The most infested fruit-square had 29 clumped larvae in the pulp, situated in the middle section. Additionally, females exhibited a highly significant preference for oviposition activities in the fruit’s shaded sections, which received three times more ovipositions than the sun-exposed portion of the fruit (Z = −3.09, *p* < 0.001; Figure 2c). The fruit with the highest number of observed ovipositions had 37 (27, 10, and 0 in upper, middle, and lower sections, respectively), and it was in the shaded area where light incidence for that fruit ranged from 530 to 610 Lux. Light-intensity on the side of the mangos that were exposed to the sun fluctuated between 362–121,373 Lux, in sharp contrast to the 238–6211 Lux measured in the shaded sides. We note however, that on cloudy days, some females did oviposit in areas otherwise exposed to sunlight on sunny days. That is, direct light incidence on the fruit had a clear effect on oviposition decisions by females.

### 3.2. Chemical Analyses of Total Polyphenols, Proteins, Lipids, and Carbohydrates

Total phenolic compounds in “Criollo” mangos not exposed to females (i.e., ovipositions) differed significantly when comparing peel and pulp (F = 1884, df = 1, 108, *p* < 0.0001), but did not differ when comparing fruit-section (upper, middle or lower) (F = 0.62, df = 2, 108, *p* = 0.54) or sunlight exposure (i.e., sun-exposed vs. shaded parts of the fruit)(F = 2.07, df = 1, 108, *p* = 0.15). Similarly, total protein concentrations were significantly different when comparing peel and pulp (F = 61.52, df = 1, 108, *p* < 0.0001), but did not differ when comparing fruit sections (F = 0.005, df = 2, 107, *p* = 0.99) or sunlight exposure (F = 0.017, df = 1, 107, *p* = 0.90).

**Figure 3:**
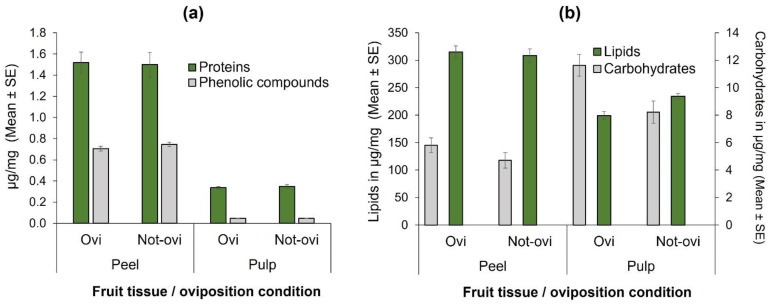
Nutrients quantified (µg/mg) according to fruit tissue (peel or pulp) and condition (with eggs [=Ovi] or free of them [Not-ovi]) in “Criollo” mango fruit: (**a**) Total phenolic compounds and total protein concentrations; (**b**) Total lipid and total carbohydrate concentrations.

When total phenolic compounds were compared in the fruit-tissue of oviposited and non-oviposited squares, no significant differences were found in neither peel (t = 1.30, df = 58, *p* = 0.20; Figure 3a) nor the pulp (t = −0.16, df = 58, *p* = 0.87; Figure 3a). Similarly, total protein concentrations did not significantly differ in the peel (t = −0.15, df = 58, *p* = 0.87; Figure 3a) and in the pulp (t = −0.71, df = 58, *p* = 0.47) of oviposited and non-oviposited fruit squares.

In the case of lipids, there were highly significant differences between pulp and peel (F = 99.58, df = 1, 101, *p* < 0.0001; Figure 3b), but not between the condition of oviposited or non-oviposited squares (F = 2.164, df = 1, 101, *p* = 0.144; Figure 3b). With respect to carbohydrates, the pattern was different (Figure 3b), as there were significantly higher concentrations in pulp than in peel (F = 46.22, df = 1, 101, *p* < 0.0001), and in oviposited than non-oviposited squares (F = 10.83, df = 1, 101, *p* < 0.001; Figure 3b).

### 3.3. Targeted Metabolomic Analysis of Fruit Phenolic Compounds

The results of the targeted metabolomic analysis of “Criollo” mangos not exposed to the oviposition activity of females, clearly indicated that the composition and concentrations of specific phenols differed significantly in peel and pulp (MANOVA: Wilks Lambda = 0.019, F = 198.27, df = “9, 36” *p* < 0.0001; Table 1), among sections of the fruit (i.e., upper, middle or lower) (MANOVA: Wilks Lambda = 0.59, F = 2.76, df = “9, 36” *p* < 0.01; Table 2), and according to incidence of sunlight (MANOVA: Wilks Lambda = 0.058, F = 2.88, df = 9, 36, *p* < 0.01; Table 2). Five major phenolic compounds were found in the peel and six in the pulp, with mangiferin and quercetin-3-D-galactoside being the only compounds that were detected in both peel and pulp (Table 1). However, when phenolic compound concentrations were individually analyzed in different tissues and sections, significant differences were found (Table 2). For example, in the peel, catechin concentration was higher in the lower section of the fruit, while quercetin and quercetin-3-D-galactoside concentrations were higher in the upper section, but in the case of pulp, no differences were detected (Table 2). With respect to light incidence, the concentration of mangiferin was higher in shaded sections of the peel, and the concentration of ferulic acid was higher in pulp stemming from sun-exposed fruit sections (Table 2).

### 3.4. Untargeted Metabolomic Analysis of Oviposited and Non-Oviposited Mangos

**Figure 4:**
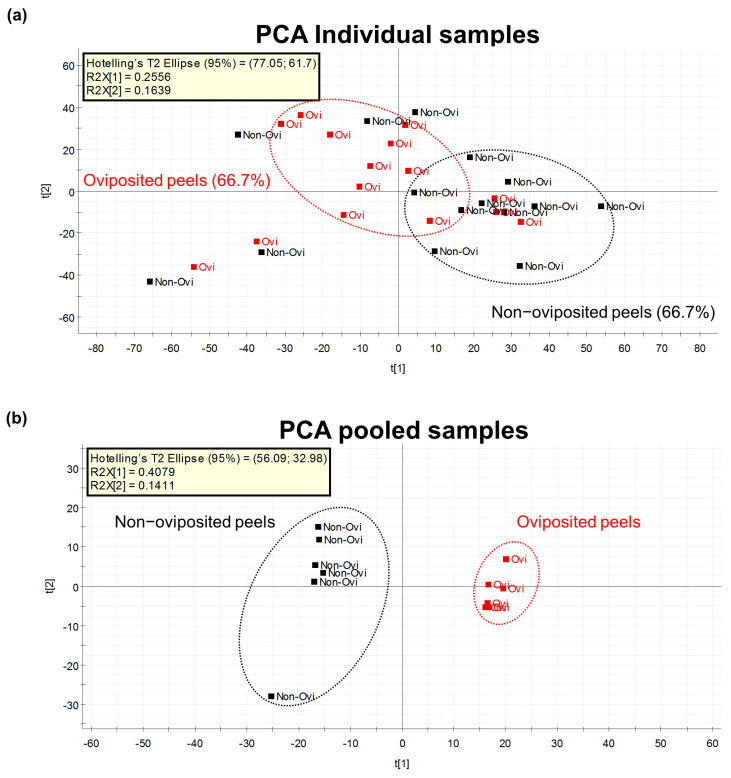
(**a**) PCA considering the metabolomic profiles detected by the untargeted metabolomics approach in individually analyzed “Criollo” mango peels into which *A. obliqua* females had inserted eggs (oviposited) or that were free of eggs (non-oviposited) (*n* = 15 per treatment); (**b**) PCA on the metabolomic profiles detected in pools of “Criollo” mango peels into which *A. obliqua* females had laid eggs (oviposited) or that were free of eggs (non-oviposited) (*n* = 6 per treatment).

**Figure 5:**
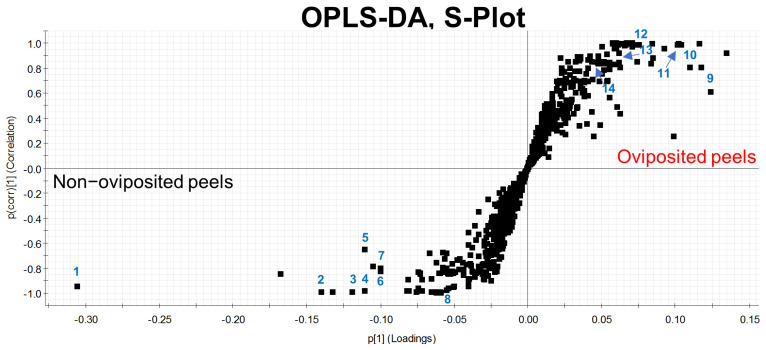
S-Plot generated through a discriminant analysis (OPLS-DA) depicting the peel of “Criollo” mangos into which *A. obliqua* females had not inserted any eggs (lower left part) versus mango peels used by females as oviposition substrates (pooled samples in both cases). The numbers indicate the position of the chemical markers shown in Table 3.

Our first Principal Component Analysis (PCA), related to our untargeted metabolomic analysis, considering individual samples of the peel into which eggs were laid and those devoid of eggs, differentiated groups in only 66.7% of samples (Figure 4a). Based on this result, we decided to prepare pooled samples from the different mango samples (oviposited and non-oviposited) and repeated the multivariate analysis. Figure 4b shows the second PCA grouping where it becomes clear that chemical profiles from peels into which eggs were laid and non-oviposited tissue were different. S-Plot (Figure 5) generated by the OPLS-DA allowed us to identify distinctive phenolic compounds as potential markers in oviposited and non-oviposited peels, such as quercetin and kaempferol/luteolin derivatives. Along with various phenolics, ABA and dihydrophaseic acid glucoside were putatively identified in higher levels in non-oviposited peels (Figure 5 and Table 3).

## 4. Discussion

We found that the specific sites in “Criollo” mangos where *A. obliqua* females lay eggs exhibit a special microenvironmental condition. Field observations under semi-natural conditions revealed that *A. obliqua* females strongly preferred to oviposit in shaded areas and upper sections of “Criollo” mangos. However, during our continuous and detailed observations, we noticed that females did oviposit in areas with sunlight incidence on cloudy days, which were equivalent to shaded areas of typical sunny days, indicating that perhaps females avoid excess heat. The observed oviposition pattern was similar to the one reported for this species in tropical plum trees (*S. mombin*) [54]. These authors reported the highest oviposition activity between 07:00 and 10:00 h when light intensity was between 32 and 215 Lux and the temperature between 27 °C and 33 °C. We note that, in areas where mangos typically grow, temperatures can exceed 40 °C [54]. Although we did not measure fruit temperature, using light intensity as a proxy of temperature instead, we suggest that females prefer to oviposit in shaded areas with lower temperatures to avoid egg mortality due to desiccation or overheating [65,66], as when eggs are inserted into the fruit, the part where the respiratory horn is located remains partially exposed [39]. We did not measure mango-skin thickness, but this is another aspect that could also explain the preference for shaded areas of the fruit by females, as mango skin is thicker in sun-exposed areas when compared to shaded ones [28]. Considering the fact that *A. obliqua* has a short ovipositor, laying eggs in the thinner peel sections of fruit will permit larvae to quickly reach deeper places in the fruit, avoiding the areas with more resin ducts which are both toxic to eggs and larvae [25,41].

The preference of *A. obliqua* females to oviposit in the upper section of fruit coincides with the preference by *B. dorsalis* females, which also prefer to lay eggs close to the peduncle [20]. These authors associated this behavior with physiological changes in mangos that generate earlier ripening and softer pericarp in upper sections of the fruit when compared with middle and bottom sections. More recently, Grechi et al. [67] working on Reunion Island with “Cogshall” mangos and the fruit flies *Bactrocera zonata* (Saunders), *Ceratitis quilicii* (Karsch) and *Ceratitis capitata* (Wiedemann), also found that infestation increased as fruit ripened. Here, we tested the fruit’s chemical quality considering that the combination of high temperature and direct sunlight impinging on the fruit over several hours can affect the homogeneous quality of the fruit [28,29] and could influence the oviposition preference of *A. obliqua* females. As in previous studies [51,68,69], our chemical analyses revealed that mango peel has significantly higher concentrations of total nutrients and specific phenolic compounds than pulp (i.e., our targeted chemical analysis; Table 1 and Table 2). We found notably higher concentrations of mangiferin, quercetin-3-D-galactoside, quercetin-3-glucoside, and quercetin in the peel than pulp.

Mango pulp contained four phenolics (protocatechuic acid, caffeic acid, vanillin, and ferulic acid) that were not present in peels (Table 2). Although the role of some phenolic compounds in plant defense is well documented [70], they can also be beneficial to animal health [71]. If we consider that “Criollo” mango is one of the best hosts of *A. obliqua,* these phenolic compounds could have positive effects on fruit fly larvae or the adults stemming from them. For example, they could possibly extend the adult lifespan, as has been shown in *Drosophila melanogaster* [72,73], or harden the cuticle of pupae and adults, in the specific case of protocatechuic acid [74,75]. However, since we found that concentrations of polyphenols in the pulp were similar throughout the fruit, with the exception of ferulic acid, it is evident that the presence of polyphenols in the pulp is not determinant in influencing oviposition site selection by *A. obliqua* females, at least in the highly susceptible mango cultivar “Criollo”. Ferulic acid, of which we measured lower concentrations in shaded fruit areas, has been reported as a phenolic compound that can have positive, negative, or neutral effects on insect development [76,77], but its role in the development of *A. obliqua* immatures has not been studied.

In the case of the fruit peel, we found significant differences in polyphenol concentrations (Table 2). Concentrations of catechin were higher in the lower sections of the fruit, unlike quercetin and quercetin 3-D-galactoside, which were in higher concentrations in the upper sections of the mangos (Table 2). Interestingly, only mangiferin, a well-known phenolic compound with potential beneficial antioxidant properties [69,78], exhibited differences in concentration with respect to sunlight exposure (Table 2). According to Léchaudel et al. [28], mangos exposed to high temperatures and intense sunlight exposure increase antioxidant levels to cope with or diminish the oxidative stress in the peel induced by UV radiation exposure. In contrast to Léchaudel et al. [28], in this study, mangiferin concentrations were higher in shaded sections of fruit peel, precisely the sites preferred by *A. obliqua* for oviposition. According to these findings, it is possible that the preference by *A. obliqua* females to oviposit in shaded areas is also influenced (on top of the incidence of direct sunlight) by the presence in higher concentrations of some beneficial compounds such as mangiferin that may positively contribute to larval development, as was demonstrated for resveratrol in *D. melanogaster* [73]. Based on a previous report [40], “Criollo” mangos are particularly suitable for the development of *A. ludens* and *A. obliqua* exhibiting shorter immature development times when compared to the resistant “Tommy Atkins” cultivar. “Criollo” mangos also have significantly thinner cuticles exhibiting higher rates of water transpiration than the resistant cultivars “Tommy Atkins” and “Kent” [41]. Reinforcing our central argument, it has been found that when fruit of the mango cultivar “Cogshall” develops in shaded areas of the tree, its epicarp (peel) is thinner when compared to fruit sections or entire fruit exposed to sunlight [28]. Furthermore, “Criollo” mangos have lower resin duct density and sap content when compared to “Tommy Atkins” mangos [25]. So, on top of avoiding laying eggs in parts of fruit directly exposed to sunlight, *A. obliqua* females could have evolved mechanisms to distinguish suitable hosts for progeny development based on the proxy detection of positive or negative cues such as sugar content or specific allelochemicals. Our chemical analyses detected that “Criollo” mangos have higher concentrations of nutrients (total proteins and lipids) and total polyphenols in the peel than in the pulp (Figure 3a,b), except for carbohydrates that were more abundant in the pulp (Figure 3b). These fruit traits could be used by females to chemically evaluate fruit quality via odors [79] or via the sensilla in the ovipositor. Further studies are needed to determine how exactly fruit flies sense the physical and biochemical factors involved in host and oviposition site selection.

Metabolomic studies based on accurate mass spectrometry represent a powerful tool that has been used to gain insights into more efficient management approaches against notorious plant pests and diseases, such as thrips [80], foraging ants [81] and fusarium wilt [82,83,84]. The untargeted metabolomics study we performed reinforces the idea that *A*. *obliqua* females can sense fruit chemical quality, as reported for *D. melanogaster* [79], since we identified biochemical differences between oviposited and non-oviposited sites in “Criollo” mangos (Figure 4b and Figure 5). Different compounds were found as potential distinguishing markers in both oviposited and non-oviposited tissues. Among the chemical markers tentatively identified in non-oviposited sites, abscisic acid (ABA) and dihydrophaseic acid glucoside, a by-product of ABA catabolism [85], stand out. ABA is involved in the fruit ripening process and in the response to biotic and abiotic stresses [86,87], including the resistance to pathogen and insect damage [88]. Considering that ABA and the juvenile hormones of insects are derived from the same precursor (farnesyl pyrophosphate) [89], and that several studies have reported different effects of ABA on insects, some authors [90] have suggested “that ABA can either act as an antagonist or an agonist of juvenile hormone signaling, depending on the ontogenetic stage and the feeding mode of the insect in question”. In Diptera, some studies report that ABA has an inhibitory effect on vitellogenesis (egg production process) and reproduction [91,92,93]. In fact, Visscher [93] patented this discovery, registering the use of ABA and its analogs (dihydrophaseic acid included) as an insect control method. In our case, the presence of ABA in non-oviposited sections suggests that females probably use it as an indicator of an unsuitable place for their eggs, but this needs to be confirmed.

Fruit ripening is strongly regulated by phytohormones, light incidence on fruit, and temperature [94]. Light influences pigment accumulation and thereby determines fruit color [94]. Thus, selecting the ideal part of the fruit to lay eggs, and then aggregating eggs in this site, is a critical component of the females optimal-foraging behavior. Díaz-Fleischer and Aluja [95] showed that in the case of *A. ludens*, females laid larger clutches in unripe fruit than in fully ripe fruit, which was related to larval survival in an unfavorable environment, as unripe fruit contain less sugar and higher concentrations of toxic allelochemicals. Here, we worked with only one degree of fruit maturity and found that while there were no differences in protein concentrations among upper and lower fruit sections, there were differences in the concentrations of specific phenolic compounds. We suggest that the observed preference of *A. obliqua* females for laying eggs in the upper fruit section may be related with the higher concentrations of beneficial polyphenols and lower concentrations of deleterious compounds in combination with a preference by the same females for particular microclimatic conditions (i.e., intensity of sunlight incidence in certain parts of the tree). Although *A. obliqua* lays only one egg per oviposition bout, during this study, we observed several ovipositions in a single fruit square (clumping), one very close to the other, which generated grouped clusters of eggs equivalent to the clutches *A. ludens* females lay. Grouping larvae has been proposed as a strategy that improves their survival as it increases metabolic heat and colonization by bacteria and yeasts that can help breakdown toxic chemicals [19]. Recently, Oroño et al. [96] reached a similar conclusion working with walnuts infested by *A. fraterculus* and *C. capitata* in Argentina. It is still unclear whether flies can sense the biochemical composition of host fruit, but our results suggest that flies might evaluate this by assessing peel quality. In *A. obliqua* [37] and *C. capitata* [97] it has been reported that females can discriminate among substrates (i.e., artificial hosts or fruit sections) with different nutritional values, selecting the substrates with higher concentrations of nutrients such as sugars or proteins to lay their eggs.

Based on what we have learned here, we deduce that exposure to sunlight is the most important factor influencing oviposition decisions by females, and that in a next step in the quality evaluation process by females, certain chemical markers, such as mangiferin concentration or ABA and dihydrophaseic acid glucoside, may be used by females to choose specific sites in the shaded parts of the fruit to select the exact location to insert the eggs. We plan additional studies to gain further insights into this critical aspect in the biology/behavior of the insect and for management purposes. In relation to the “preference-performance” and “mothers know best” hypotheses, we suggest that *A. obliqua* females can indeed recognize the fruit parts with better conditions for the development of eggs and larvae. As a next step, we plan a detailed study on the sensilla in the proboscis, vertex, maxillary palps, and aculeus tip, which apparently help females in assessing the quality of the potential oviposition site. Once a gravid female lands on fruit, it walks over its surface exhibiting continuous head buttings (i.e., the female lowers its head and repeatedly touches the surface of the fruit with sensilla that are found in the vertex) until it locates a site into which it inserts its aculeus, sometimes retrieving it (probing) or to lay eggs, while at the same time extruding the proboscis [53]. Therefore, assessing if the sensilla located in the proboscis, maxillary palps, vertex, and aculeus tip respond to mangiferin, ABA and dihydrophaseic acid glucoside or the other compounds we identified in the skin and pulp of “Criollo” mangos (or yet to be identified), is necessary if we are to advance in our quest to fully understand what exactly drives *A. obliqua* (or *A. ludens*) female oviposition decisions. Further research on this is also necessary using other mango cultivars that are more tolerant to the attack of *A. obliqua* and *A. ludens*, such as “Ataulfo”, “Tommy Atkins”, “Kent”, “Edward” and “Brooks Late” [40].

## 5. Conclusions

**Figure 6:**
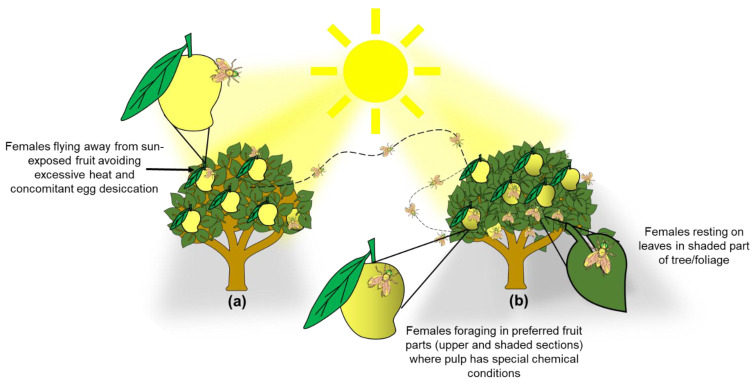
Graphical depiction of trees exhibiting two conditions to illustrate the management scheme proposed based on our findings and on Aluja et al. [104]. (**a**) Pruned tree with fewer branches/foliage to enhance sunlight incidence in the inner parts of the canopy to promote desiccation of *A. obliqua* eggs in the peel of mango fruit and force females to seek out trees with more foliage and benign environmental conditions; (**b**) Tree with excessive branching/foliage which generates ideal conditions for *A. obliqua* female foraging and egg laying (fruit infestation) (details at the end of Discussion).

Our results have direct implications for the management of *A. obliqua* in commercial mango orchards and backyard gardens. Clearly, a pruning scheme that exposes most fruit to direct sunlight [2] would be highly effective, as it is known that the epidermis (skin) of sun-exposed fruit is thicker (e.g., [28]) and, as documented here, females avoid sun-exposed fruit. Furthermore, if eggs are laid in such fruit, desiccation would kill them, because as we mentioned before, often when eggs are inserted into the fruit, the part where the respiratory horn is located remains partially exposed [39]. In this sense, using mango cultivars with more sap in the fruit such as “Ataulfo”, and “Tommy Atkins” [25] will also help block the respiratory horn of *A. obliqua* eggs. According to this information, managing this pest and other fruit flies that also attack mango fruit could be more efficient if most of the actions to control females are focused on disturbing their preferred places for oviposition activities (Figure 6). Taking advantage of these aspects by exposing most fruit to direct sunlight and the concomitant heat, would inhibit many females from laying eggs, and if they did, most eggs would die through desiccation. In such a management scheme, it could be advisable to leave a couple of larger, unpruned trees per row or in strategic locations of the orchard to attract females to the more benign microenvironment, serving as egg sinks. That is, a certain amount of fruit would be “sacrificed” and culled, but most fruit would remain uninfested. If, on top of this, a host marking pheromone (HMP) is applied to pruned trees with sun-exposed fruit [9,98], *A. obliqua* and *A. ludens* adults would be forced out of HMP-treated trees and would infest the fruit in the trees with more benign environmental conditions. This takes advantage of the fact that there is interspecific cross-recognition of the HMP between the two *Anastrepha* species (*A*. *obliqua* and *A*. *ludens*) that attack mango in Mexico [98,99]. In the unpruned trees, traps or bait stations [100,101] can also be placed to kill the attracted flies. If the orchard is not too big or we are dealing with a backyard garden, bagging fruit would be a highly efficient alternative [102,103].

## Figures and Tables

**Table 1:** Polyphenol concentrations in mango peel and pulp resulting from the “targeted metabolomic analysis of plant phenolic compounds” in fruit not exposed to female oviposition activity.

Phenolic Compounds	Peel Mean ± SE µg/g of Dried Sample	Pulp Mean ± SE µg/g of Dried Sample
(+)-Catechin	77.18 ± 7.29	0
Mangiferin	580.44 ± 25.94	0.65 ± 0.21
Quercetin	16.17 ± 1.42	0
Quercetin-3-D-galactoside	317.21 ± 24.10	1.70 ± 0.11
Quercetin-3-glucoside	313.82 ± 28.36	0
Protocatechuic acid	0	0.46 ± 0.01
Caffeic acid	0	0.38 ± 0.02
Vanillin	0	0.44 ± 0.01
Ferulic acid	0	0.63 ± 0.06

**Table 2:** Concentrations of polyphenols and significance values of factorial ANOVAs of peel and pulp polyphenols considering sunlight incidence and fruit section (upper vs. lower) as factors from “targeted metabolomic analysis of plant phenolic compounds study” in mangos not exposed to female oviposition activity. Data reported as the mean ± SE (µg/g).

Peel Polyphenols	Sunny Side Mean ± SE		Shaded Side Mean ± SE		Significant Values			
					Fruit Section		Sunlight Incidence	
	Upper Section	Lower Section	Upper Section	Lower Section	F	*p*	F	*p*
Catechin	41.12 ± 6.66	97.84 ± 12.84	66.97 ± 12.16	102.79 ± 11.07	9.38	**0.001**	1.98	0.17
Mangiferin	527.59 ± 33.92	512.49 ± 49.89	664.77 ± 41.45	616.9 ± 62.08	0.27	0.76	6.33	**0.02**
Quercetin	20.18 ± 3.85	12.66 ± 1.34	19.43 ± 2.07	12.42 ± 2.32	4.01	**0.03**	0.036	0.85
Quercetin-3-D-galactoside	407.15 ± 60.98	258.91 ± 13.74	353.95 ± 37.39	248.83 ± 43.33	4.60	**0.02**	0.56	0.46
Quercetin-3-glucoside	337.81 ±86.63	269.10 ± 24.21	385.30 ± 44.04	263.10 ±52.17	1.54	0.23	0.13	0.72
**Pulp Polyphenols**	**Sunny Side** **Mean ± SE**		**Shaded Side** **Mean ± SE**		**Significant Values**			
					**Fruit Section**		**Sunlight Incidence**	
	**Upper Section**	**Lower Section**	**Upper Section**	**Lower Section**	**F**	***p***	**F**	***p***
Caffeic acid	0.40 ± 0.04	0.43 ± 0.05	0.35 ± 0.01	0.35 ± 0.01	0.20	0.820	3.28	0.084
Ferulic acid	0.78 ± 0.09	0.81 ± 0.11	0.54 ± 0.11	0.39 ±0.13	0.45	0.643	8.92	**0.007**
Mangiferin	1.38 ± 0.75	0.62 ± 0.28	0.20 ± 0.06	0.41 ± 0.19	0.92	0.41	2.82	0.108
Protocatechuic acid	0.47 ± 0.02	0.47 ± 0.03	0.42 ± 0.04	0.47 ± 0.02	0.69	0.512	1.14	0.296
Quercetin-3-D-galactoside	1.99 ± 0.39	1.68 ± 0.15	1.36 ± 0.12	1.76 ± 0.16	1.11	0.347	1.38	0.252
Vanillin	0.43 ± 0.02	0.46 ± 0.03	0.41 ± 0.04	0.46 ± 0.02	0.98	0.38	0.16	0.688

**Table 3:** Tentative identification of the main differential chemical markers found in “Criollo” mango peel from “untargeted metabolomic analysis” of upper and shaded mango peel sections into which *A. obliqua* females were forced to lay eggs into or that were devoid of eggs.

Condition	Peak	Rt	*m*/*z*	Formula	Ion	Error	Tentative Identification	Fragments	
**Non-oviposited**	1	0.47	381.0797	C_12_H_22_O_11_K^+^	[M+K]^+^	−0.5	Disaccharide fragment	365.1052	203.0521
	2	3.91	771.1033	C_32_H_28_O_21_^+^	[M+Na]^+^	1.6	Pentose-Quercetin derivative	457.0742	303.0503
	3	0.39	251.0310	C_13_H_8_O_4_Na^+^	[M+Na]^+^	−4.0	Dihydroxyxanthone derivative	203.0379	185.0282
	4	2.02	577.1190	C_32_H_28_O_21_^+^	[M+H]^+^	−0.5	Kaempferol/luteolin derivative	287.0553	153.0178
	5	4.2	923.1141	C_36_H_36_O_26_^+^	[M+K]^+^	1.0	Quercetin derivative	409.1103	303.0501
	6	3.16	467.1889	C_21_H_32_O_10_Na^+^	[M+Na]^+^	−0.9	Dihydrophaseic acid glucoside	265.1442	-
	7	4.01	449.1783	C_21_H_30_O_9_Na^+^	[M+Na]^+^	−1.1	Quercetin derivative	303.0500	153.0174
	8	5.26	287.1259	C_15_H_20_O_4_Na^+^	[M+Na]^+^	0.0	Abscisic acid	247.1346	229.1245
**Oviposited**	9	3.74	303.0502	C_15_H_11_O_7_^+^	[M+H]^+^	−1.0	Quercetin-galactoside	487.0850	153.0182
	10	2.34	577.1187	C_26_H_25_O_15_^+^	[M+H]^+^	−1.0	Kaempferol/Luteolin derivative	287.0553	153.0178
	11	1.1	268.1′43	C_10_H_14_O_4_N_5_^+^	[M+H]^+^	−1.1	Adenosine	136.0613	-
	12	3.28	575.1044	C_26_H_23_O_15_^+^	[M+H]^+^	1.2	Mangiferin gallate	285.0419	257.0452
	13	3.37	619.1241	C_26_H_28_O_16_Na^+^	[M+Na]^+^	−5.5	Quercetin derivative	303.0494	153.0186
	14	2.85	315.0703	C_13_H_15_O_9_^+^	[M+H]^+^	−4.1	Gallic acid derivative	153.0183	-

## Data Availability

Experimental data are available on request from the corresponding authors.
